# Supplementary material for: Association between oral microbiome and seven types of cancers in East Asian population: a two-sample Mendelian randomization analysis
Source: Front Mol Biosci. 2023 Nov 21;10:1327893. doi: 10.3389/fmolb.2023.1327893 (PMC10702768; doi:10.3389/fmolb.2023.1327893)
Supplement: Supplementary file 1 [file DataSheet1.ZIP › Supplementary Materials/MR plots of saliva/saliva═╝/Breast cancer/pheno.2055_to_breast cancer_forest.pdf]

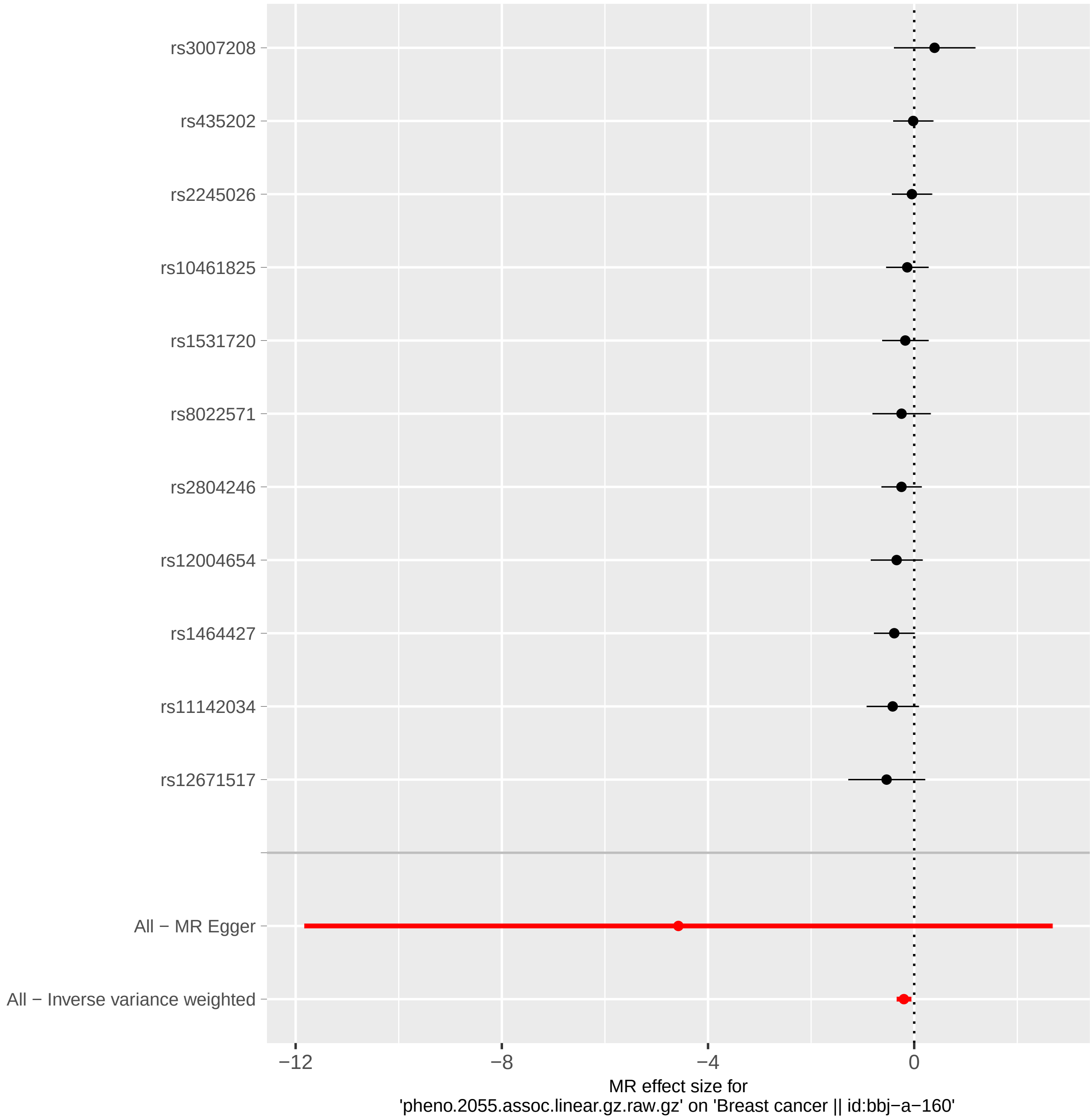
rs3007208
rs435202
rs2245026
rs10461825
rs1531720
rs8022571
rs2804246
rs12004654
rs1464427
rs11142034
rs12671517
All − MR Egger
All − Inverse variance weighted
−12
−8
−4
0
MR effect size for
'pheno.2055.assoc.linear.gz.raw.gz' on 'Breast cancer || id:bbj−a−160'
